# Stressors and Resources for Healthcare Professionals During the Covid-19 Pandemic: Lesson Learned From Italy

**DOI:** 10.3389/fpsyg.2020.02179

**Published:** 2020-10-08

**Authors:** Serena Barello, Lorenzo Palamenghi, Guendalina Graffigna

**Affiliations:** ^1^EngageMinds HUB - Consumer, Food & Health Engagement Research Center, Università Cattolica del Sacro Cuore, Milan, Italy; ^2^Department of Psychology, Università Cattolica del Sacro Cuore, Milan, Italy; ^3^Faculty of Psychology, Università Cattolica del Sacro Cuore, Milan, Italy; ^4^Faculty of Agriculture, Food and Environmental Sciences, Università Cattolica del Sacro Cuore, Piacenza, Italy

**Keywords:** burnout, distress, healthcare professionals, COVID-19, job demands

## Abstract

The COVID-19 pandemic is exerting a high pressure on healthcare systems all over the world. Italy, in particular, being one of the first Western countries to be struck by the contagion, has seen the number of recovered -and deceased- patients increase alarmingly, thus increasing the workload and the demands for healthcare professionals. This situation has the potential to put several healthcare operators at risk of developing high levels of work-related distress and burnout due to the exposure to emotionally difficult situations, uncertainty, and personal risk. A sample of 532 Italian physicians, nurses, and other professionals answered an online survey addressing their levels of burnout (through the Maslach Burnout Inventory) and frequency of experienced psycho-somatic symptoms, along with some *ad hoc* items regarding job demands. Results show that levels of burnout and experienced symptoms are correlated with the increased demands due to the COVID-19 pandemic, while finding a meaning in one’s own work is correlated with personal gratification. Urgent measures to address concerns regarding the wellbeing of health workers are a necessary key point of the response to the current pandemic.

## Introduction

The COVID-19 pandemic has disrupted healthcare systems worldwide, unlike anything else in the last few decades: during the emergency, operating rooms have been transformed into ICUs, healthcare professionals of many different backgrounds have been drafted into emergency work, and many of them have contracted the disease as well.

This scenario has been experienced internationally, although some countries such as Italy were particularly overwhelmed (Armocida et al., 2020; Nacoti et al., 2020). Since Feb 21, 2020, when the first case of COVID-19 was recorded in Italy, the National Healthcare Service, which offers universal access to health care, has faced increasing pressure, with 231,732 total assessed cases of COVID-19 and 33,142 deaths as of May 28th, 2020 (Ministero della Salute, 2020). In the most affected regions, the National Healthcare Service almost collapsed, as mechanical ventilators, oxygen, and personal protective equipment were not available for everyone. And as with any event of this magnitude, COVID-19 will not just cause many victims, but will also take its toll in terms of the psychological burden that those who survive will have to bear (Holmes et al., 2020).

This “emotional surge” has the potential to burden the medical workforce for as long as the public health crisis lasts (Downar and Seccareccia, 2010). Healthcare professionals found themselves working at the front line of the COVID-19 outbreak response and as such are exposed to several risks for their own occupational safety and psycho-physical health (Lima et al., 2020). Indeed, they experienced unprecedented psychological and physical symptoms of grief in response to patients’ suffering and death (Li et al., 2020; Barello et al., 2020a). They have been exposed to traumatic events and situations that could lead to significant distress and moral suffering (Delfrate et al., 2018; Barello and Guendalina, 2020; Radbruch et al., 2020; Barello et al., 2020b), such as difficult triage decisions regarding the allocation of limited resources to the patients that they are personally taking care of Selman et al. (2020). All of these potentially traumatic experiences have occurred under extreme pressures, including the fear of spreading the virus to loved ones, possible separation from family, mental and physical exhaustion, and limited access to personal protective equipment and medical supplies. Although not all healthcare workers are going to develop mental health problems, no one is invulnerable or immune, and some healthcare staff will struggle, possibly for an extended time, as they face unprecedented and unexpected scenarios.

A pandemic causes and amplifies suffering through physical illness, death, stresses, and anxieties that the entire healthcare workforce is currently facing across multiple countries (Adams and Walls, 2020). Therefore, the response to this pandemic should be based on key attributes such as supporting complex decision-making and managing medical uncertainty (Williamson et al., 2020); however, this implies that the current emergency may actually challenge the medical culture, its implicit assumptions, and the basic underpinnings of daily work.

According to this premises, there is an urgent need to mitigate the psycho-social impact of the COVID-19 pandemic on healthcare workers to address broader aspects of wellbeing among them. Hence, recognizing the sources of work-related stress is required for healthcare organizations to develop targeted approaches and to address concerns and provide specific support to their health care workforce.

Understanding the stressors that COVID-19 is placing on Italian clinicians, their perceptions about job demands and job resources, and their impact on physical and mental health can assist in recognizing what is needed to return to a point of wellness during and after such emergencies.

Therefore, this study was aimed to (1) describe the levels of burnout of a sample of Italian healthcare workers involved in the management of the COVID-19 pandemic and to (2) explore the relationship between professionals’ burnout and psychosomatic symptoms with perceived job demands and job resources.

## Methods

A group of 744 Italian healthcare professionals was asked to answer a survey regarding their burnout levels and their experience at work during the COVID-19 outbreak. Of these, 532 provided complete answers between the 4th and the 27th of April, 2020. Table 1 shows sample characteristics.

**TABLE 1:** Personal and professional sample characteristics.

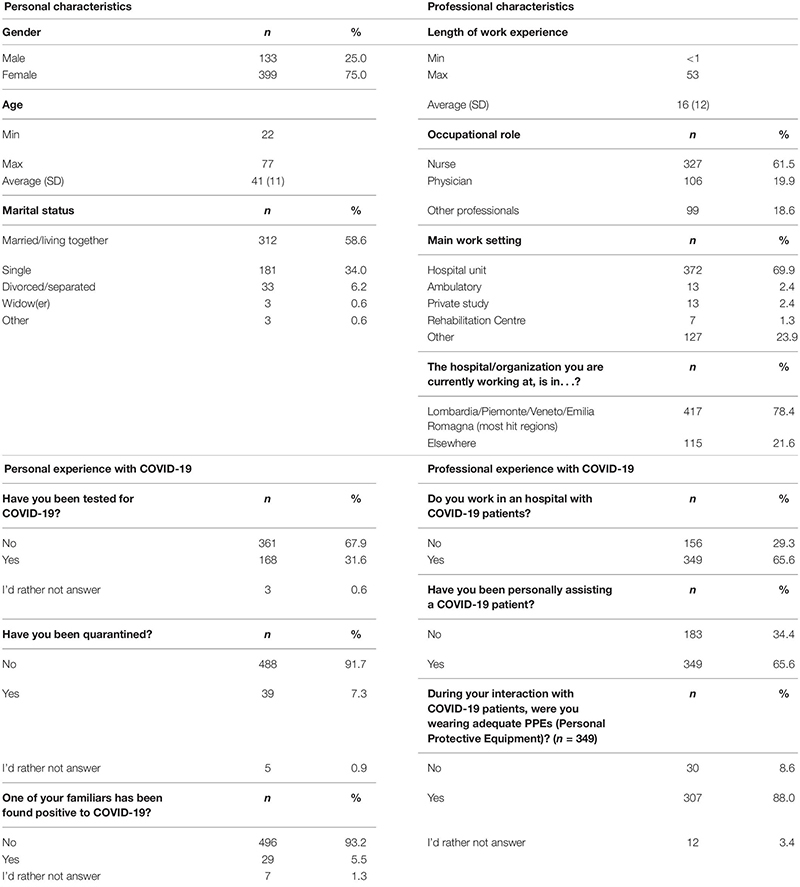

The survey included the Maslach Burnout Inventory (Maslach et al., 1996), a 22 items questionnaire, considered the gold standard for burnout assessment, which provides 3 different indexes of burnout of healthcare operators (Emotional Exhaustion, Depersonalization, and Personal Gratification). The survey also included a series of questions regarding the perceived job demands and resources (in particular: professional risks, emotional demands, uncertainty, work-family balance, and meaning of work). Finally, our survey comprised a checklist of psycho-somatic symptoms that could have been experienced by healthcare professionals under heavy workloads and distress: participants were asked to report the frequency of these symptoms in the last 4 weeks on a 6-point scale from “never” to “usually.” The answers were then averaged to calculate an index of “psycho-somatic distress.” All participants provided written informed consent and the study was approved by the Catholic University Ethical Commission (approval number 2020–04).

## Results

Our results show that, in our sample of Italian healthcare professionals, levels of burnout were high: according to the Italian cut-off criteria for healthcare workers (Sirigatti and Stefanile, 1993), 41% showed high levels of Emotional Exhaustion, and 27% high levels of Depersonalization, while only 57% were really gaining high levels of gratification from their own work. Generally speaking, the COVID-19 pandemic was demanding a high toll from Italian healthcare professionals: 91.8% of the sample agreed with the statement that “the COVID-19 emergency puts me more frequently in touch with other people’s suffering,” while 70.6% agreed with the statement “My job is putting me at serious risk.”

A series of Spearman’s correlations was run to assess the association between burnout levels, psycho-somatic distress, and job demands to better understand the factors underlying these high levels of burnout and distress. Table 2 shows correlation indexes.

**TABLE 2:** Spearman’s correlations between professional demands and indexes of burnout/distress^a^.

	Emotional exhaustion	Depersonalization	Personal gratification	Psycho-somatic distress
**Professional risks**				
My job is putting me at serious risk	0.360 (*p* < 0.001)	0.172 (*p* < 0.001)		0.358 (*p* < 0.001)
The health risk caused by my job is unacceptable	0.332 (*p* < 0.001)	0.160 (*p* < 0.001)		0.303 (*p* < 0.001)
**Emotional demands**				
The COVID-19 emergency makes me take difficult decisions at work	0.244 (*p* < 0.001)			0.233 (*p* < 0.001)
I often feel like I need to hide my emotions at work	0.292 (*p* < 0.001)	0.096 (*p* = 0.027)		0.420 (*p* < 0.001)
At work I usually do things I don’t want to	0.364 (*p* < 0.001)	0.228 (*p* < 0.001)	−0.143 (*p* = 0.001)	0.299 (*p* < 0.001)
The COVID-19 emergency puts me more frequently in touch with other people’s suffering	0.139 (*p* = 0.001)		0.096 (*p* = 0.28)	0.231 (*p* < 0.001)
**Uncertainty**				
I have difficulty at tolerating the unpredictability of the COVID-19 emergency	0.284 (*p* < 0.001)	0.108 (*p* = 0.013)		0.341 (*p* < 0.001)
I cannot tolerate the uncertainty of curing COVID-19 patients	0.302 (*p* < 0.001)	0.110 (*p* = 0.013)		0.368 (*p* < 0.001)
**Work-family balance**				
My private life is being affected by the energies I’m spending at work	0.396 (*p* < 0.001)	0.146 (*p* = 0.001)		0.336 (*p* < 0.001)
Since the COVID-19 emergency has begun, I cannot pass enough time with my family	0.260 (*p* < 0.001)	0.125 (*p* = 0.004)		0.277 (*p* < 0.001)
**Meaning of work**				
At work, I can fully express myself	−0.344 (*p* < 0.001)	−0.330 (*p* < 0.001)	0.429 (*p* < 0.001)	−0.117 (*p* < 0.001)
My job is inspiring	−0.316 (*p* < 0.001)	−0.280 (*p* < 0.001)	0.435 (*p* < 0.001)	−0.123 (*p* = 0.005)

*^*a*^Non-significant correlations (*p* > 0.05) have not been reported.*

In particular, health professionals’ perceived levels of professional risk, emotional demands, uncertainty of the clinical situation, and conflict between work and family were correlated with the experience of burnout and, in particular, with emotional exhaustion. They were also correlated with the frequency of psycho-somatic symptoms, while they did not seem correlated with personal gratification.

On the other hand, the ability to feel that one’s own work has a meaning and to be inspired by the work was negatively correlated with both emotional exhaustion and depersonalization, while positively correlated with personal gratification.

## Discussion

The current COVID-19 pandemic is not only having a direct impact on citizens and economy but also, and particularly, on the healthcare system and professionals’ health in Italy. As the National Healthcare System was trying to keep up with the growing number of cases, healthcare professionals were asked to comply with increasingly difficult-to-face challenges, higher job demands and increased workload, which eventually interfered with their private life and work-family balance. Moreover, emotional demands increased as well, as healthcare professionals found themselves more frequently facing other people’s sufferings, complicated decisions, and uncertain situations on top of severe risks for their own health. Our findings show that the perception of these increased demands is indeed associated with the levels of burnout we observed in our sample (in particular, with emotional exhaustion) and with the frequency of experienced symptoms that could be indices of psycho-somatic distress.

This is coherent with scientific literature exploring the levels of burnout and distress among healthcare professionals that, even in their “routine” experience, are requested to face complicated decisions, heavy emotional loads and other people’s suffering with a high frequency. Indeed, physicians, nurses, and other non-specialists in this field are known to experience high levels of burnout and distress due to the very high demands that their job requests (Harrison et al., 2017; Rizo-Baeza et al., 2018).

In this situation, the capacity of the professionals to find a meaning in their work, and to be inspired by it, seems to act as an important resource and a protective factor, as higher levels are associated with less emotional exhaustion and depersonalization and with higher personal gratification at work. Thus, according to previous studies on this topic (West et al., 2018), while reducing workloads, providing adequate protective equipment and psychological support are crucial strategies to reduce the current levels of burnout, finding a way to support and enhance work motivation could be essential in preventing or limiting burnout and other distress-related health outcomes.

Therefore, we suggest that strategies to support healthcare professionals, such as peer-to-peer counseling, self-monitoring and pacing, working in teams, and organizational supervision to support professionals’ motivation at work and mitigate the impact of continued exposure to death and dying, emotional exhaustion, desperation, and suffering should be urgently deployed across health systems worldwide. To enable clinicians to maintain personal well-being and resilience throughout the pandemic, healthcare organizations should aim to monitor both clinician sources of stress and to sustain their personal work motivation and work engagement. These efforts are warranted to proactively address concerns related to the wellbeing of clinicians and their families. Alleviation of healthcare professionals’ suffering needs to be a key part of the strategic response to the COVID-19 pandemic.

This study has a few limitations, in particular regarding generalizability, as the sample is not statistically representative of the Italian population of healthcare workers. Moreover, future cross-cultural studies should study the psychological impact of COVID-19 on healthcare workers in other countries and cultures for comparison.

## Data Availability Statement

The raw data supporting the conclusions of this article will be made available by the authors, without undue reservation.

## Ethics Statement

The studies involving human participants were reviewed and approved by Catholic University Ethical Commission. The patients/participants provided their written informed consent to participate in this study.

## Author Contributions

SB and GG equally contributed to the research conceptualization and methodology. SB and LP cured draft writing and manuscript preparation. LP cured data and carried out formal analyses. GG supervised the work. All authors approved the final manuscript.

## Conflict of Interest

The authors declare that the research was conducted in the absence of any commercial or financial relationships that could be construed as a potential conflict of interest.

## References

[B1] Adams J. G. Walls R. M. (2020). Supporting the health care workforce during the COVID-19 global epidemic. *JAMA - J. Am. Med. Assoc.* 323 1439–1440. 10.1001/jama.2020.3972 32163102

[B2] Armocida B. Formenti B. Ussai S. Palestra F. Missoni E. (2020). The Italian health system and the COVID-19 challenge. *Lancet Public Health* 5:e253 10.1016/s2468-2667(20)30074-8 PMC7104094 32220653

[B3] Barello S. Guendalina G. (2020). Caring for health professionals in the COVID-19 pandemic emergency: towards an “epidemic of empathy” in healthcare. *Front. Psychol.* 11:1431. 10.3389/fpsyg.2020.01431 32581986 PMC7296111

[B4] Barello S. Palamenghi L. Graffigna G. (2020a). Burnout and somatic symptoms among frontline healthcare professionals at the peak of the Italian COVID-19 pandemic. *Psychiatry Res.* 290:113129. 10.1016/j.psychres.2020.113129 32485487 PMC7255285

[B5] Barello S. Palamenghi L. Graffigna G. (2020b). Empathic communication as a “Risky Strength” for Health during the COVID-19 pandemic: the case of frontline italian healthcare workers. *Pat. Educ. Couns.* 103 2200–2202. 10.1016/j.pec.2020.06.027 32631648 PMC7313503

[B6] Delfrate F. Ferrara P. Spotti D. Terzoni S. Lamiani G. Canciani E. (2018). Moral Distress (MD) and burnout in mental health nurses: a multicenter survey. *Med. del Lav* 109 97–109. 10.23749/mdl.v109i2.6876 PMC7682177 29701626

[B7] Downar J. Seccareccia D. (2010). Palliating a Pandemic: “All Patients Must Be Cared For.”. *J. Pain Symptom Manage* 39 291–295. 10.1016/j.jpainsymman.2009.11.241 20152591 PMC7135517

[B8] Harrison K. L. Dzeng E. Ritchie C. S. Shanafelt T. D. Kamal A. H. Bull J. H. (2017). Addressing palliative care clinician burnout in organizations: a workforce necessity, an ethical imperative. *J. Pain Symptom Manage* 53 1091–1096. 10.1016/j.jpainsymman.2017.01.007 28196784 PMC5474199

[B9] Holmes E. A. O’Connor R. C. Perry V. H. Tracey I. Wessely S. Arseneault L. (2020). Multidisciplinary research priorities for the COVID-19 pandemic: a call for action for mental health science. *Lancet Psychiatry* 7 547–560. 32304649 10.1016/S2215-0366(20)30168-1 PMC7159850

[B10] Li Z. Ge J. Yang M. Feng J. Qiao M. Jiang R. (2020). Vicarious traumatization in the general public, members, and non-members of medical teams aiding in COVID-19 control. *Brain Behav. Immun.* 88 916–919. 10.1016/j.bbi.2020.03.007 32169498 PMC7102670

[B11] Lima C. K. T. Carvalho P. M. de M. Lima I. de Aas, Nunes J. V. A. (2020). The emotional impact of Coronavirus 2019-nCoV (new Coronavirus disease). *Psychiatry Res.* 287:112915. 10.1016/j.psychres.2020.112915 32199182 PMC7195292

[B12] Maslach C. Jackson S. E. Leiter M. P. (1996). *Maslach Burnout Inventory Manual.* California, CA: Consulting Psychologists Press.

[B13] Ministero della Salute (2020). *Covid-19, Situation Report Update at 28 May 18.00.* Rome: Ministero della Salute.

[B14] Nacoti M. Ciocca A. Giupponi A. Brambillasca P. Lussana F. Pisano M. (2020). At the epicenter of the Covid-19 pandemic and humanitarian crises in Italy: changing perspectives on preparation and mitigation. *Catal non-issue Content.* Online ahead of print.

[B15] Radbruch L. Knaul F. M. de Lima L. de Joncheere C. Bhadelia A. (2020). The key role of palliative care in response to the COVID-19 tsunami of suffering. *Lancet* 395 1467–1469. 10.1016/s0140-6736(20)30964-8 32333842 PMC7176394

[B16] Rizo-Baeza M. Mendiola-Infante S. V. Sepehri A. Palazón-Bru A. Gil-Guillén V. F. Cortés-Castell E. (2018). Burnout syndrome in nurses working in palliative care units: an analysis of associated factors. *J. Nurs. Manag.* 26 19–25. 10.1111/jonm.12506 28695723

[B17] Selman L. E. Chao D. Sowden R. Marshall S. Chamberlain C. Koffman J. (2020). Bereavement support on the frontline of COVID-19: recommendations for hospital clinicians. *J. Pain Symptom Manage* 60 e81–e86. 32376262 10.1016/j.jpainsymman.2020.04.024 PMC7196538

[B18] Sirigatti S. Stefanile C. (1993). *MBI - Maslach Burnout Inventory. Adattamento e Taratura per l’Italia.* Firenze: Organizzazioni Speciali, 33–42.

[B19] West C. P. Dyrbye L. N. Shanafelt T. D. (2018). Physician burnout: contributors, consequences and solutions. *J. Int. Med.* 283 516–529. 10.1111/joim.12752 29505159

[B20] Williamson V. Murphy D. Greenberg N. (2020). COVID-19 and experiences of moral injury in front-line key workers. *Occup. Med. (Lond).* 70 317–319. 10.1093/occmed/kqaa052 32239155 PMC7184422

